# Analysis of the efficacy of upfront brain radiotherapy versus deferred radiotherapy for EGFR/ALK-positive non-small cell lung cancer with brain metastases: a retrospective study

**DOI:** 10.1186/s12885-024-11868-9

**Published:** 2024-01-23

**Authors:** Jing Qian, Zelai He, Ying Wu, Hongwei Li, Qun Zhang, Xianming Li

**Affiliations:** 1 Jinan University, Guangzhou, Guangdong China; 2 Department of Radiation Oncology, The First Affiliated Hospital of Bengbu Medical University, Bengbu, Anhui China; 3 Department of Radiation Oncology, The 2nd Clinical Medical College (Shenzhen People’s Hospital) of Jinan University, Shenzhen, Guangdong China

**Keywords:** EGFR, ALK, Non-small cell lung cancer, Radiotherapy, Brain metastases

## Abstract

**Background:**

For brain metastases (BMs) from *EGFR/ALK*-positive non-small cell lung cancer (NSCLC), the best time to administer tyrosine kinase inhibitors (TKIs) and brain radiotherapy (RT) has not been identified. This analysis was an attempt to solve this problem in part.

**Methods:**

A total of 163 patients with *EGFR/ALK*-positive NSCLC and brain metastasis (BM) who were diagnosed between January 2017 and July 2022 were included in this study. Ninety-one patients underwent upfront RT, and 72 patients received deferred RT. Comparing the clinical efficacy and safety in these two patient cohorts was the main goal of the study.

**Results:**

The average follow-up period was 20.5 months (range 2.0 to 91.9 months). The median overall survival (OS) was 26.5 months, and the median intracranial progression-free survival (iPFS) was 23.6 months. Upfront RT considerably increased the iPFS (26.9 vs. 20.2 months, hazard ratio [HR] = 5.408, *P* = 0.020) and OS (31.2 vs. 22.3 months, HR = 4.667, *P* = 0.031) compared to deferred RT. According to multivariate analysis, upfront RT was an independent risk factor for predicting iPFS (HR = 1.670, *P* = 0.021). Upfront RT (HR = 1.531, *P* = 0.044), TKI therapy (HR = 0.423, *P* < 0.001), and oligometastases (HR = 2.052, *P* = 0.021) were found to be independent risk factors for OS.

**Conclusion:**

This study showed that upfront RT combined with TKI treatment can significantly improve intracranial disease management and prolong survival in patients with *EGFR/ALK* mutations in BMs from NSCLC.

**Supplementary Information:**

The online version contains supplementary material available at 10.1186/s12885-024-11868-9.

## Introduction

The most common cause of brain metastasis (BM) is NSCLC [1]. Less than 5% of patients with advanced or metastatic disease will survive for more than five years, making them at low risk of success [2]. Systemic therapy, particularly chemotherapy, has substantial limitations for treating BM because of the poor permeability of the blood‒brain barrier (BBB) and low intracranial response rate [3–5]. Traditionally, the main treatments for BM are whole-brain radiotherapy (WBRT), stereotactic radiosurgery (SRS), or surgery. However, WBRT may impair neurocognitive abilities and lower patients’ quality of life [6]. Similar survival rates and reduced neurotoxicity are linked to SRS, which usually affects a restricted number and size of BMs [7, 8]. With the discovery of critical mutations necessary for the growth and spread of tumours, particularly those involving the epidermal growth factor receptor (EGFR) and anaplastic lymphoma kinase (ALK), platinum-based chemotherapy has given way to a new era of molecular targeted therapy for the treatment of NSCLC [9, 10]. Approximately 30–40% of Asian advanced NSCLC patients are positive for *EGFR* mutations [11]. A greater percentage of patients with *EGFR* mutations experience BM than patients with wild-type EGFR [12]. Research has demonstrated that NSCLC patients with *EGFR* mutations have a superior response to RT. There is a theoretical basis for combination therapy comprising RT and EGFR-tyrosine kinase inhibitors (TKIs) since RT disrupts the BBB and increases drug permeability. In contrast, TKIs can proportionally increase the radiosensitivity of EGFR-mutant cells [13]. The *EML4/ALK* fusion gene is an uncommon gene that can activate ALK tyrosine kinase and downstream pathways. It is found in 3–7% of NSCLCs [14]. In NSCLC, ALK inhibitors, particularly second- and third-generation inhibitors, have shown promising treatment results [15]. For NSCLC patients with BM, brain RT and TKIs are currently the primary therapeutic options, and they are both productive and safe. There is no precise intervention timing advice regarding the administration of brain RT or TKIs. In earlier research, most patients consented to first-generation TKIs; however, there is ongoing debate on the widespread use of second- or third-generation TKIs and whether they can postpone the need for RT.

Therefore, our goal was to investigate the best time to combine TKIs with brain RT by examining the BM data of EGFR/ALK-positive NSCLC patients at our centre.

## Materials and methods

### Patient selection

**Fig. 1:**
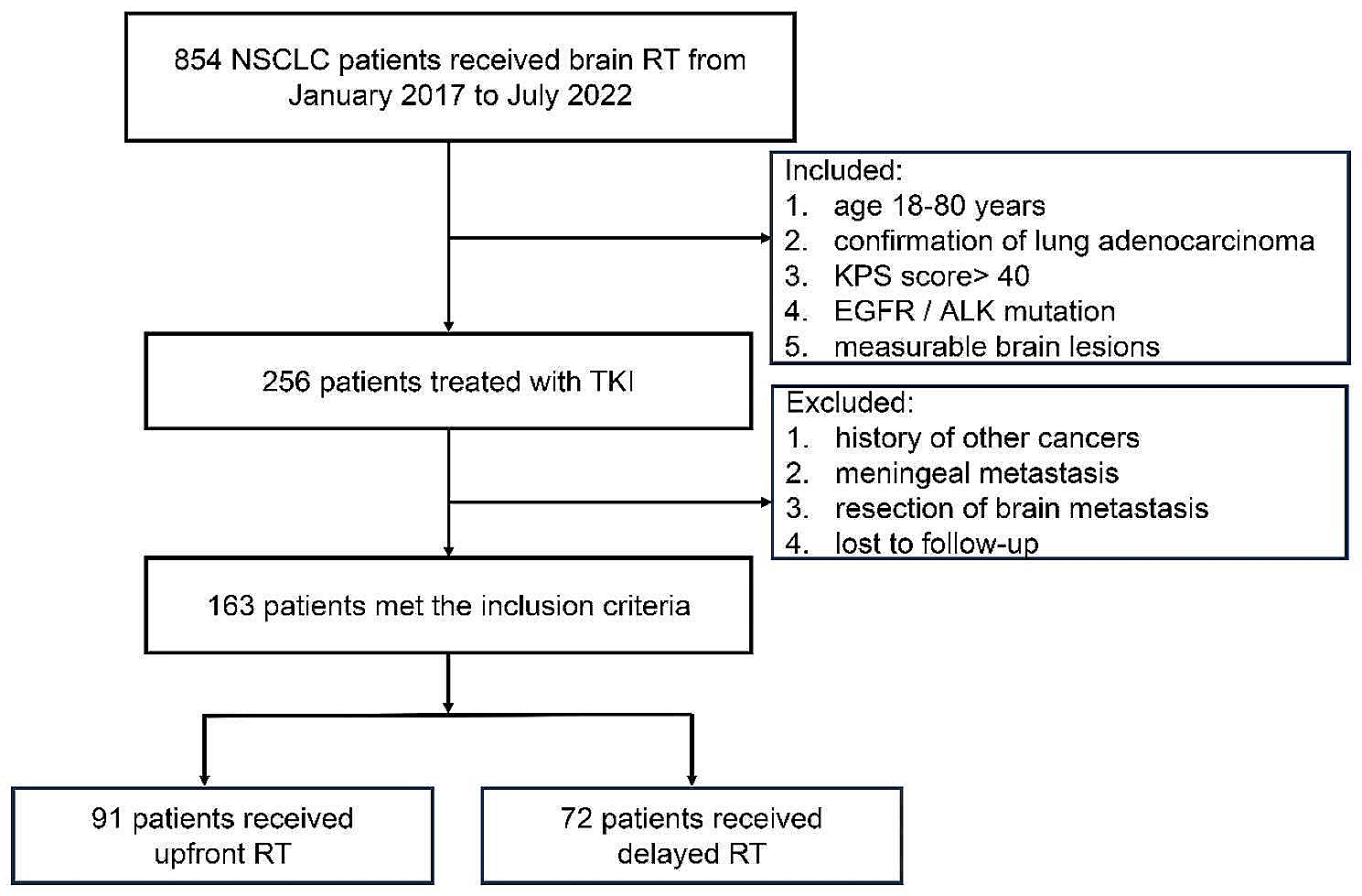
Flow diagram depicting the inclusion process of the study participants

From January 2017 to July 2022, we enrolled 163 patients with driver-positive NSCLC with BM at the First Affiliated Hospital of Bengbu Medical University who met the eligibility requirements as shown in Fig. 1. The following conditions were part of the enrolment criteria: (1) age ranging from 18 to 80 years; (2) confirmation of lung adenocarcinoma; (3) Karnofsky Performance Status (KPS) score greater than 40; (4) *EGFR/ALK* mutation; and (5) magnetic resonance imaging (MRI) or enhanced computed tomography (CT) was used to identify quantifiable brain lesions. The exclusion criteria were as follows: (1) history of other cancers; (2) presence of meningeal metastasis upon BM diagnosis; (3) brain metastasis excision; and (4) loss to follow-up.

### Treatment strategies

WBRT, local radiotherapy (LCRT), and WBRT + LCRT were the three types of brain RT. The prescribed WBRT dose was generally 30 Gy in 10 fractions or 40 Gy in 20 fractions, ranging from 30 to 45 Gy in 10–20 fractions. The prescribed LCRT dose ranged from 35 to 60 Gy in 5–27 fractions. In the WBRT + LCRT group, LCRT included sequential and simultaneous boosts. The prescribed dose of the sequential boost ranged from 45 to 60 Gy in 15 to 30 fractions. The simultaneous boost prescribed dose ranged from 40 to 56 Gy in 10 to 20 fractions.

There were 147 patients with *EGFR* mutations in total. The first-line treatment for 100 patients was first-generation EGFR-TKIs (gefitinib, *n* = 71; icotinib, *n* = 22; and erlotinib, *n* = 7). Fifty-six patients were switched from first-generation to third-generation TKIs after receiving first-generation TKIs. The remaining 47 patients were given third-generation EGFR-TKIs (osimertinib, *n* = 41; almonertinib, *n* = 6). Sixteen patients had ALK arrangements; one received crizotinib, nine received crizotinib and then switched to alectinib, five received alectinib, and one received lorlatinib.

### Outcome measurement

Patients usually received follow-up evaluations every three months, which could include enhanced MRI of the brain, chest CT, abdominal ultrasound, and, if needed, bone scans or positron emission tomography. The response rate was assessed using the Response Evaluation Criteria in Solid Tumours (version 1.1) by at least two radiologists with experience. The period between BM and the patient’s death or the final follow-up was used to compute overall survival (OS). The time interval between BM and intracranial progression or the last follow-up was used to calculate intracranial progression-free survival (iPFS). The total complete response (CR) and partial response (PR) for intracranial reactions was the intracranial objective response rate (iORR).

### Statistical analysis

With the Statistical Package for Social Sciences 26.0, a statistical analysis of the data was carried out. Classification count data were represented using the number of patients (%) and the χ2 test to compare the two cohorts. The log-rank test and Kaplan‒Meier survival analyses were used to compare OS with iPFS. The factors affecting OS and iPFS were analysed using a Cox proportional hazards model. Every test level was considered to be statistically significant at *P* < 0.05.

## Results

### Patient characteristics

This study included 163 patients in total; 91 patients (55.8%) had upfront RT, and 72 (44.2%) had deferred RT. A total of 144 patients (88.3%) underwent brain MRI, and 19 patients (11.7%) underwent enhanced CT to evaluate intracranial lesions. There were 63 males (38.7%) and 100 females (61.3%). With a median age of 59 (ranging from 26 to 79 years), the average age was 58.6 years. One hundred seventeen patients (71.8%) had intracranial symptoms, while 100 patients (61.3%) had a KPS score ≥ 80. One hundred two patients (62.6%) received a diagnosis of BM. WBRT was given to 63 patients (38.7%), LCRT was given to 54 patients (33.1%), and WBRT + LCRT was given to 46 patients (28.2%). Twenty patients (12.2%) in the WBRT + LCRT group received sequential boost, and 26 patients (16.0%) received simultaneous boost. The lung-molGPA dataset included age, number of BMs, KPS score, extracranial metastases and gene mutation type and is used to predict the prognosis of NSCLC patients with BM. In 79 patients (48.5%), the lung-molGPA was 1 to 2.5; in 84 patients (51.5%), it was 3 to 4. *EGFR* mutations were found in 147 patients (90.2%), 60 of whom (36.8%) had 19 exons, 67 of whom (41.1%) had 21 exons, and 20 of whom (12.2%) had uncommon or unknown mutations. Sixteen patients (9.8%) had *ALK* mutations. Forty-two patients (25.8%) received only first-generation TKIs, and 121 patients (74.2%) received second- or third-generation TKIs. Seventy-two patients (44.2%) were oligometastatic, and 91 patients (55.8%) were polymetastatic. Extracranial metastases were present in 73 patients (44.8%). Seventy-eight patients (47.9%) had a BM diameter ≥ 2 cm, while 85 patients (52.1%) had a maximal BM diameter of less than 2 cm. Among the patients, 77 (47.2%) had a BM number ≤ 3, and 86 (52.8%) had a BM number greater than 3. Except for initial BM and extracranial metastases, the characteristics of the two cohorts were essentially balanced (*P* > 0.05). Table 1 displays the essential clinicopathological characteristics of the patients.

**Table 1:** Baseline characteristics of patients

Characteristics	Upfront RT(*n* = 91) N (%)	Deferred RT(*n* = 72) N (%)	χ^2^	*P*
Sex			1.538	0.215
Male	39(42.9)	24(33.3)		
Female	52(57.1)	48(66.7)		
Age			0.835	0.361
< 60	44(48.4)	40(55.6)		
≥ 60	47(51.6)	32(44.4)		
KPS			2.446	0.118
< 80	40(44.0)	23(31.9)		
≥ 80	51(56.0)	49(68.1)		
Symptomatic BM			0.012	0.911
NO	26(28.6)	20(27.8)		
YES	65(71.4)	52(72.2)		
Initial BM			30.910	< 0.001
NO	17(18.7)	44(61.1)		
YES	74(81.3)	28(38.9)		
RT pattern			2.766	0.251
WBRT	31(34.0)	32(44.4)		
LCRT	30(33.0)	24(33.3)		
WBRT + LCRT	30(33.0)	16(22.3)		
Lung-molGPA			0.001	0.974
1-2.5	44(48.4)	35(48.6)		
3–4	47(51.6)	37(51.4)		
Gene-mutation type			1.050	0.306
EGFR mutation	84(92.3)	63(87.5)		
19	41(45.0)	19(26.4)		
21	37(40.7)	30(41.7)		
others	6(6.6)	14(19.4)		
ALK rearrangements	7(7.7)	9(12.5)		
Generation of TKI			2.695	0.101
First generation	28(30.8)	14(19.4)		
Second and third generation	63(69.2)	58(80.6)		
Metastases			2.328	0.127
Oligometastases	45(49.5)	27(37.5)		
Polymetastases	46(50.5)	45(62.5)		
Extracranial disease			4.590	0.032
NO	57(62.6)	33(45.8)		
YES	34(37.4)	39(54.2)		
BM diameter			1.189	0.275
< 2 cm	44(48.4)	41(56.9)		
≥ 2 cm	47(51.6)	31(43.1)		
BM number			2.508	0.113
≤ 3	48(52.7)	29(40.3)		
> 3	43(47.3)	43(59.7)		

### Univariate and multivariate analysis

Univariate analysis (Table 2) revealed a relationship between iPFS and extracranial metastasis incidence (HR = 1.557, 95% CI = 1.010–2.400; *P* = 0.045) as well as between iPFS and treatment regimen (deferred RT vs. upfront RT, HR = 1.651, 95% CI = 1.076–2.532; *P* = 0.022). Upfront RT could significantly increase the iPFS of patients, as multivariate analysis revealed that upfront RT was an independent risk factor for predicting iPFS (HR = 1.571, 95% CI = 1.020–2.418; *P* = 0.040).

**Table 2:** Univariate and multivariate analyses of clinical factors associated with iPFS.

Variables	Univariable Analysis				Multivariable Analysis		
	HR (95% CI)	Waldχ^2^	*P*		HR (95% CI)	Waldχ^2^	*P*
Sex							
Female vs. female	0.854(0.547 ~ 1.333)	0.485	0.486		-	-	-
Age							
≥ 60 vs. <60	1.246(0.810 ~ 1.917)	1.004	0.316		-	-	-
KPS							
≥ 80 vs. <80	0.740(0.476 ~ 1.150)	1.791	0.181		-	-	-
Symptomatic BM							
YES vs. NO	1.073(0.654 ~ 1.763)	0.078	0.779		-	-	-
Initial BM							
YES vs. NO	0.846(0.546 ~ 1.312)	0.558	0.455		-	-	-
RT pattern		2.449	0.294				
WBRT	1.000				-	-	-
LCRT	1.163(0.706 ~ 1.917)	0.353	0.552		-	-	-
WBRT + LCRT	0.753(0.433 ~ 1.31)	1.007	0.316		-	-	-
Lung-molGPA							
3–4 vs. 1-2.5	0.685(0.444 ~ 1.056)	2.931	0.087		0.819(0.486 ~ 1.380)	0.562	0.454
Gene-mutation type							
ALK vs. EGFR	1.292(0.644 ~ 2.593)	0.521	0.471		-	-	-
Generation of TKI							
Second and third vs. First	0.768(0.468 ~ 1.260)	1.089	0.297				
Metastases							
Poly vs. Oligometastases	1.322(0.859 ~ 2.035)	1.610	0.204				
Extracranial disease							
YES vs. NO	1.557(1.010 ~ 2.400)	4.028	0.045		1.304(0.769 ~ 2.210)	0.972	0.324
BM diameter							
≥ 2 cm vs. <2 cm	0.861(0.561 ~ 1.322)	0.467	0.495		-	-	-
BM number							
> 3 vs. ≤3	1.059(0.690 ~ 1.624)	0.069	0.793		-	-	-
Treatment regimen							
deferred RT vs. upfront RT	1.651(1.076 ~ 2.532)	5.277	0.022		1.571(1.020 ~ 2.418)	4.210	0.040

Univariate analysis (Table 3) revealed that OS was related to sex (HR = 0.674, 95% CI: 0.459–0.991, *P* = 0.045), the use of TKIs (HR = 0.547, 95% CI: 0.363–0.825, *P* = 0.004), metastatic status (HR = 2.247, 95% CI: 1.500 ~ 3.365, *P* < 0.001), extracranial metastases (HR = 1.632, 95% CI: 1.115–2.388, *P* = 0.012), BM number (HR = 1.621, 95% CI: 1.100–2.389, *P* = 0.015) and treatment regimen (deferred RT vs. upfront RT, HR = 1.518, 95% CI: 1.036–2.224, *P* = 0.032). Multivariate analysis also revealed that second- and third-generation TKIs could significantly improve OS compared to first-generation TKIs (HR = 0.423, *P* < 0.001). The OS of patients with oligometastases was significantly better than that of patients with polymetastases (HR = 2.052, *P* = 0.021). Upfront RT increased OS compared to deferred RT (HR = 1.531, *P* = 0.044).

**Table 3:** Univariate and multivariate analyses of clinical factors associated with OS

Variables	Univariable Analysis				Multivariable Analysis		
	HR (95% CI)	Waldχ^2^	*P*		HR (95% CI)	Waldχ^2^	*P*
Sex							
Female vs. female	0.674(0.459 ~ 0.991)	4.031	0.045		0.691(0.463 ~ 1.030)	3.294	0.070
Age							
≥ 60 vs. <60	1.147(0.781 ~ 1.683)	0.487	0.485		-	-	-
KPS							
≥ 80 vs. <80	0.852(0.579 ~ 1.256)	0.652	0.419		-	-	-
Symptomatic BM							
YES vs. NO	0.837(0.55 ~ 1.275)	0.687	0.407		-	-	-
Initial BM							
YES vs. NO	1.023(0.687 ~ 1.523)	0.013	0.911		-	-	-
RT pattern		5.083	0.079			3.508	0.173
WBRT	1.000				1.000	-	-
LCRT	0.672(0.424 ~ 1.064)	2.875	0.090		1.020(0.569 ~ 1.827)	0.004	0.947
WBRT + LCRT	0.616(0.384 ~ 0.988)	4.047	0.044		0.655(0.399 ~ 1.074)	2.818	0.093
Lung-molGPA							
3–4 vs. 1-2.5	0.847(0.579 ~ 1.240)	0.726	0.394		-	-	-
Gene-mutation type							
ALK vs. EGFR	0.646(0.283 ~ 1.475)	1.076	0.300		-	-	-
Generation of TKI							
Second and third vs. First	0.547(0.363 ~ 0.825)	8.296	0.004		0.423(0.268 ~ 0.666)	13.796	< 0.001
Metastases							
Poly vs. oligometastases	2.247(1.500 ~ 3.365)	15.427	< 0.001		2.052(1.115 ~ 3.777)	5.335	0.021
Extracranial disease							
YES vs. NO	1.632(1.115 ~ 2.388)	6.360	0.012		1.135(0.701 ~ 1.836)	0.265	0.607
BM diameter							
≥ 2 cm vs. <2 cm	0.805(0.548 ~ 1.183)	1.222	0.269		-	-	-
BM number							
> 3 vs. ≤3	1.621(1.100 ~ 2.389)	5.952	0.015		0.974(0.545 ~ 1.741)	0.008	0.928
Treatment regimen							
deferred RT vs. upfront RT	1.518(1.036 ~ 2.224)	4.591	0.032		1.531(1.011 ~ 2.318)	4.055	0.044

### Survival analysis and failure pattern

**Fig. 2:**
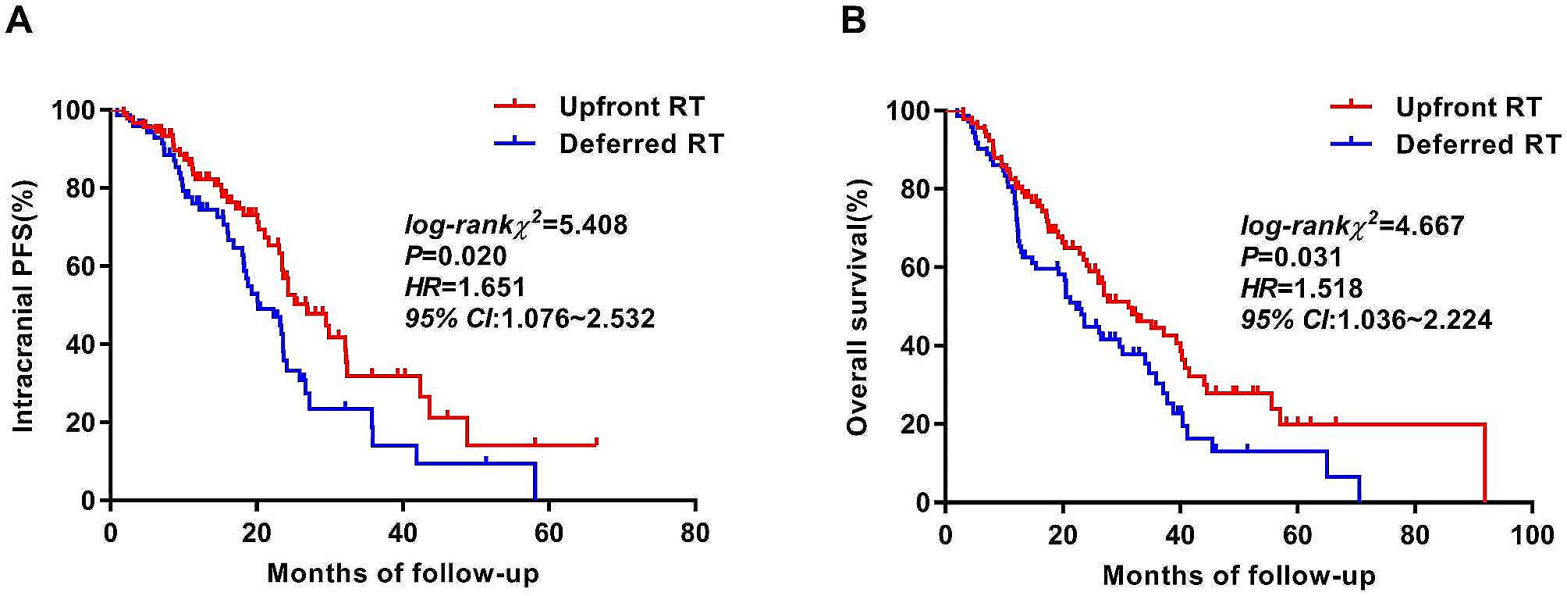
(**A**) iPFS between the upfront RT and deferred RT groups; (**B**) OS between the upfront RT and deferred RT groups

The follow-up period as of July 2022 was 20.5 months (median), with a range of 2.0 to 91.9 months. The median iPFS and OS were 23.6 months and 26.5 months, respectively. Patient iPFS (26.9 vs. 20.2 months, HR = 5.408, *P* = 0.020) and OS (31.2 vs. 22.3 months, HR = 4.667, *P* = 0.031) were significantly prolonged by upfront RT compared with deferred RT (Table 4). Figure 2 displays the Kaplan‒Meier curves for iPFS and OS.

**Table 4:** Survival statistics for the two treatment cohorts [median (95% CI)]

	Upfront RT	Deferred RT	Log-rank	*P*
iPFS	26.90(20.38,33.42)	20.20(15.15.25.25)	5.408	0.020
OS	31.20(22.24,40.16)	22.30(18.96,25.64)	4.667	0.031

**Fig. 3:**
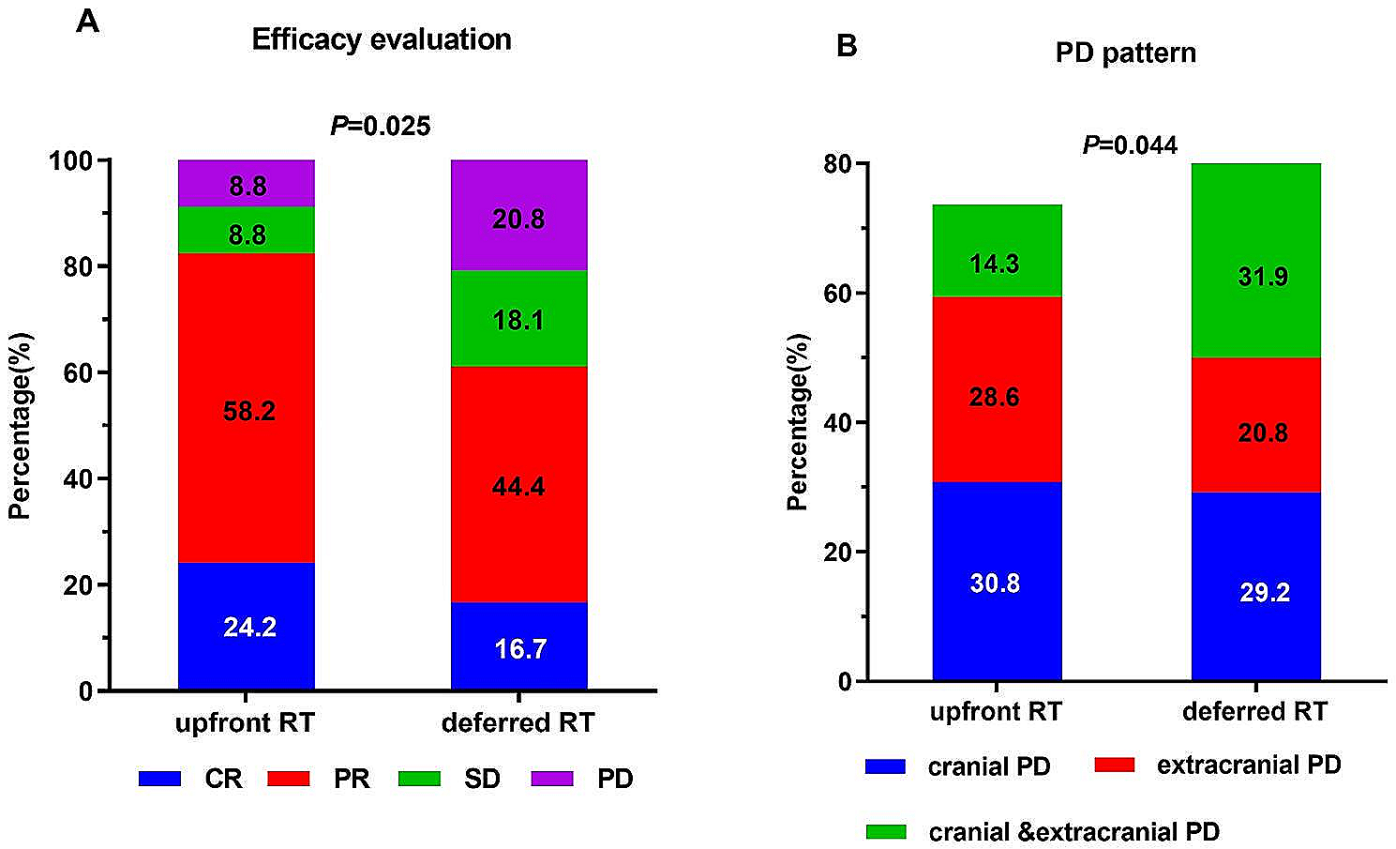
(**A**) efficacy evaluation after RT; (**B**) pattern of treatment failure

Compared to 12 patients (16.7%) who achieved CR and 32 patients (44.4%) who achieved PR in the deferred RT group, 22 patients (24.2%) achieved CR, and 53 patients (58.2%) achieved PR in the upfront RT group. The iORR of patients was considerably increased by upfront RT (82.4% vs. 61.1%, *P* = 0.025) (Fig. 3A).

Progressive disease (PD) affected 67 patients (73.6%) in the upfront RT group and 59 patients (81.9%) in the deferred RT group at the time of follow-up. In the upfront and deferred groups, there were 28 (30.8%) and 21 (29.2%) patients with intracranial PD, 26 (28.6%) and 15 (20.8%) patients with extracranial PD, and 13 (14.3%) and 23 (31.9%) patients with concurrent intracranial and extracranial PD, respectively. The upfront RT group exhibited a lower proportion of patients with intracranial PD (45.1% vs. 61.1%, *P* = 0.044) than the deferred RT group (Fig. 3B).

As of follow-up, 108 patients (66.3%) had passed away, with 91 patients (55.8%) having died of PD disease. Fourteen patients died of pulmonary infection and cachexia, one patient died of a myocardial infarction, and two patients died of pulmonary embolism.

### Toxicities

The most common acute toxic reactions included rash, decreased appetite, and neutropenia. The majority of hazardous reactions fell into the Grade 2 category. Among the 27 (16.6%) patients who experienced rash, 3 (1.8%) had a grade 3 rash and stopped taking their medicine. Grade 3 bone marrow suppression occurred in three patients (1.8%). Following RT, 23 patients (14.1%) had varying degrees of memory and cognitive impairment; only two patients (1.2%) had radiation-induced brain necrosis. No deaths linked to treatment were documented.

## Discussion

Lung cancer often manifests as BM, particularly in patients with driver-positive NSCLC. After three years, the rates were 46.7% and 58.4%, respectively, for patients with *EGFR* mutations and patients with *ALK* rearrangements who had BM at the time of initial diagnosis (24.4% and 23.8%, respectively) [13]. According to previous studies, integrating brain RT with TKI treatment improves intracranial disease management and OS more than using TKIs alone [9, 16]. Nevertheless, the best time to integrate RT with TKIs is debated.

In NSCLC patients with BM, we found that upfront RT significantly increased the median iPFS (26.9 vs. 20.2 months, *P* = 0.020) and OS (31.2 vs. 22.3 months, *P* = 0.031), and both regimens had good tolerability. This was in contrast to postponed RT. Moreover, the iORR in the upfront RT group (82.4% vs. 61.1%, *P* = 0.025) was significantly greater than that in the deferred RT group. We believe that upfront RT improves intracranial control, leading to OS advantages. Like our findings, a plethora of research has demonstrated that upfront RT enhances iPFS and OS and increases the rate of BM remission. A retrospective study of 198 eligible patients revealed a significant improvement in iPFS with upfront RT (19.9 vs. 11.1 months, *P* < 0.001) [17]. According to Magnuson et al., there were significant differences in OS (46 vs. 30 vs. 25 months, *P* = 0.001) and iPFS (23 vs. 24 vs. 17 months, *p* = 0.025) between patients treated with SRS + TKI, WBRT + TKI, or TKI [16]. On the other hand, several studies have shown no appreciable benefit of upfront RT administration over deferred RT. Brain RT plus TKIs enhanced OS compared to TKIs alone in an important analysis of 571 patients. However, there was no correlation between the duration of RT and OS (21.8 vs. 25.0 months, *P* = 0.500) [9].

Most clinical studies define oligometastases as involvement of fewer than 3 metastatic organs and fewer than 5 metastatic lesions [18]. Patients with oligometastases often survive longer than those with polymetastases. Compared to individuals with many metastases, patients with oligometastatic BMs have longer survival times, milder cognitive impairment, and fewer symptoms affecting the central nervous system. In our study, the OS of oligometastatic patients was significantly better than that of patients with polymetastases (HR = 2.052, 95% CI = 1.115–3.777, *P* = 0.021), consistent with previous reports. The number of BMs is often considered an important prognostic factor [19]. According to a recent study, OS improved only in patients with wild-type phenotypes who had fewer BMs (*P* = 0.006), whereas the number of BMs in the *EGFR/ALK*-positive group did not affect OS (*P* = 0.740) [20]. Univariate analysis in our study revealed a relationship between OS and BM number (HR = 1.621, 95% CI = 1.100–2.389, *P* = 0.015), while multivariate analysis revealed no link. Additional investigations are necessary to fully explore the effects of the quantity of BM.

Research has revealed that brain RT, particularly WBRT, can negatively impact neurocognitive function and cause neurotoxicity, which can be quite damaging to patients [6]. Although SRS is comparatively less dangerous than WBRT, it is nevertheless linked to radiation necrosis and can result in severe neurological side effects such as chronic headaches and limb weakness [7, 21]. In this study, although the proportion of patients with multiple BMs was greater in the WBRT group, there was no difference in iPFS or OS between patients in the WBRT, LCRT, or WBRT + LCRT groups. However, the WBRT group included more patients with multiple BMs. These findings indicated that while WBRT is an effective treatment modality, it is inappropriate for patients receiving LCRT. In our study, 58.4% of patients with a BM number ≤ 3 underwent LCRT, and 58.1% of patients with a BM number > 3 received WBRT. Only 1.2% of patients had radiation-induced brain necrosis, while 14.1% of patients overall had memory and cognitive impairment to varying degrees following RT.

Next-generation TKIs are thought to improve PFS and OS more than first-generation equivalents for patients with driver-positive NSCLC [22–25]. For *EGFR*-mutated NSCLC, the FLAURA study compared osimertinib with erlotinib and gefitinib. These patients showed improvements in PFS (18.9 vs. 10.2 months), a reduction in the incidence of PD in the central nervous system (6% vs. 15%), and an extension of OS (38.6 vs. 31.8 months) [26]. In a worldwide, multicentre, randomized, phase 3 trial, the 1-year PFS rates in the lorlatinib and crizotinib groups were 78% and 39%, respectively (*P* < 0.001), and the corresponding iORRs were 76% and 58%, respectively [22]. In our study, 25.8% of patients received only first-generation TKIs, 74.2% of patients received second- or third-generation TKIs, and second- and third-generation TKIs significantly improved OS (HR = 0.423, *P* < 0.001). However, additional investigations are needed to determine whether next-generation TKIs that penetrate the central nervous system can be used as standalone treatments without first requiring RT.

There are several drawbacks to the present study. This was a retrospective study, and the results may have been affected on that basis. The study also had a small sample size, bias in baseline characteristics, and a greater prevalence of first BM and extracranial metastases in the upfront RT group. A prospective, large-scale, multicentre trial is expected to shed further light on the question of when to combine brain RT and TKIs.

## Conclusion

This study demonstrated that in patients with *EGFR* or *ALK* mutations in BMs from NSCLC, upfront RT in combination with TKIs dramatically enhanced intracranial control and prolonged survival. While a small percentage of patients will experience modest cognitive impairment and memory loss, most people will tolerate treatment well.

### Electronic supplementary material

Below is the link to the electronic supplementary material.

Supplementary Material 1

## Data Availability

The raw data can be accessed in the Supplementary File.
